# Influence of single nucleotide polymorphisms among cigarette smoking and non-smoking patients with coronary artery disease, urinary bladder cancer and lung cancer

**DOI:** 10.1371/journal.pone.0243084

**Published:** 2021-01-28

**Authors:** Nongnit Laytragoon Lewin, Jan-Erik Karlsson, David Robinsson, Matida Fagerberg, Magnus Kentsson, Shariel Sayardoust, Mats Nilsson, Levar Shamoun, Bengt-Åke Andersson, Sture Löfgren, Lars Erik Rutqvist, Freddi Lewin

**Affiliations:** 1 Dept Laboratory Medicine, Ryhov Hospital, Jönköping, Sweden; 2 Dept of Internal Medicine, Ryhov Hospital, Jönköping, Sweden; 3 Dept of Health Medicine and Caring Sciences, Linköping University, Linköping, Sweden; 4 Dept Urology, Ryhov Hospital, Jönköping, Sweden; 5 Dept Periodontology, Ryhov Hospital, Jönköping, Sweden; 6 Futurum, Academy of Health and Care, Region Jönköping, Jönköping, Sweden; 7 Dept Medical and Health Sciences, Linköping University, Linköping, Sweden; 8 Scientific Affairs Group, Swedish Match AB, Stockholm, Sweden; 9 Dept Oncology, Ryhov Hospital, Jönköping, Sweden; King Saud University, SAUDI ARABIA

## Abstract

**Introduction:**

Cigarette smoke is suggested to be a risk factor for coronary artery disease (CAD), urinary bladder cancer (UBCa) or lung cancer (LCa). However, not all heavy smokers develop these diseases and elevated cancer risk among first-degree relatives suggests an important role of genetic factor.

**Methods:**

Three hundred and ten healthy blood donors (controls), 98 CAD, 74 UBCa and 38 LCa patients were included in this pilot study. The influence of 92 single nucleotide polymorphisms (SNPs) and impact of cigarette smoking were analysed.

**Results:**

Out of 92 SNPs tested, differences in distribution of 14 SNPs were detected between controls and patient groups. Only *CTLA4* rs3087243 showed difference in both CAD and UBCa patient group compared to control group. Stratified by smoking status, the impact of smoking was associated to frequencies of 8, 3 and 4 SNPs in CAD, UBCa, LCa patients, respectively. None of these 92 SNPs showed a statistically significant difference to more than one type of disease among smoking patients. In non-smoking patients, 7, 3 and 6 SNPs were associated to CAD, UBCa, LCa, respectively. Out of these 92 SNPs, *CTLA4* rs3087243 was associated to both non-smoking CAD and UBCa. The *XRCC1 r*s25487 was associated to both non-smoking UBCa and LCa.

**Conclusion:**

SNPs might be important risk factors for CAD, UBCa and LCa. Distribution of the SNPs was specific for each patient group, not a random event. Impact of cigarette smoking on the disease was associated to the specific SNP sequences. Thus, smoking individuals with SNPs associated to risk of these serious diseases is an important target group for smoking cessation programs.

## Introduction

Cigarette smoke is a toxic and carcinogenic agent that is suggested to be a major risk factor for serious diseases [1, 2]. The smoking associated diseases could among other diseases, be coronary artery disease (CAD), head and neck cancer, urinary bladder cancer (UBCa), obstructive pulmonary diseases or lung cancer (LCa) [3–6].

It is assumed that about 50% of all tobacco smokers will die from smoking and 60% of all deaths caused by cigarette smoke are in cancer or CAD [7, 8]. The mechanisms that determines which one of the smoking related disease each patient will suffer from are unknown. Smoking related diseases put a major strain on the health care systems and are major cause of early death in the world [8].

However, not all heavy smokers develop tobacco induced diseases. Elevated cancer risk found among first-degree relatives of cancer patients suggests an important role of genetic factors [9, 10]. Single nucleotide polymorphism (SNP) is the most common source of human genetic variation in DNA sequences [11–13]. SNPs are inborn and lifelong stable. SNPs might influence risk of individual specific disease independents from cigarette smoke toxic agents.

Cigarette smoke induces massive normal cell death in vitro [13]. As a consequence of massive normal cell death, long term cigarette smoking could induce systemic chronic inflammation and immune-suppression in healthy smokers [4]. The possible impact of genetics and cigarette smoking on circulating immune response cells and inflammatory biomarkers was also found in healthy smokers [4, 14]. Overtime, a chronic inflammatory environment might influence tumor suppressor genes, oncogenes and various functional genes [15, 16].

In this pilot study, the SNP distribution in CAD, UBCa, LCa patients were compared with healthy controls, and the impact of cigarette smoking on these diseases were investigated. Ninety-two SNPs in genes associated to cell cycle, cell death, immune response, DNA repair, inflammation, microRNA and oncogenesis were analyzed.

## Material and methods

### Patients and controls

A total of 512 individuals were investigated. The study patients (n = 210) and controls (n = 302) were from a community-based population of European descent in Jönköping region, Sweden (Table 1). A non-randomized and discretionary group of CAD, UBCa and LCa patients, aged ≥ 19 years were invited to participate. No power calculation was applied since this was a pilot study aimed to be hypothesis generating. The patient inclusion criteria were based on relevant diagnostic procedure or pathological diagnosis of the given diseases.

**Table 1:** Characteristics of coronary artery disease (CAD), urinary bladder cancer (UBCa), lung cancer (LCa) patients and healthy blood donors (controls).

		Mean aged, year (SD)	Sex (n)		Smoking (n)	
	n		Male	Female	Yes	No
**Controls**	**302**	**56 (11)**	**155**	**147**	**142**	**160**
**Patients**						
**CAD**	**98**	**68(9)**	**83**	**15**	**55**	**43**
**UBCa**	**74**	**74(9)**	**59**	**15**	**15**	**59**
**LCa**	**38**	**67(8)**	**12**	**26**	**31**	**7**
**Total**	**512**		**309**	**203**	**243**	**269**

Healthy controls, aged ≥ 19 years were recruited from the blood bank, the periodontal clinic, and the smoking prevention clinic. Samples from the periodontal clinic donors were drawn at least three months after any treatment and the individuals showed no clinical signs of local inflammation. None of the controls had a history of cardiovascular disease, kidney disorder, malignant or pulmonary disease.

Thirty ml peripheral blood was collected from the controls and patients using EDTA containing tubes. All blood samples were stored at -80°C in the biobank, at Ryhov hospital, Sweden until analysed.

### Ethics statement

This pilot study was conducted in accordance with the Declaration of Helsinki and Regional Research Ethics Review Board of Linköping, Sweden approved our study (Dnr 2011/271-31 and 2015/178-32). Written informed consent was obtained from all participants.

### SNP analysis

High molecular weight DNA was extracted from blood samples using QIAGEN Bio Robot M48 with MagAttract DNA Blood M48 kits EZ1 (www.qiagen.com). The quantity and quality of DNA was determined by NanoDrop. According to genetics home reference-NIH (https://ghr.nlm.nih.gov/gene) and previous investigations, 92 SNPs located in genes associated to cell cycle controls, cell death, immune response, DNA repair, inflammation, microRNAs, oncogenes and tumour suppressor genes (Table 2) were selected for this study [4, 12–14, 17, 18].

**Table 2:** Genes and SNPs.

Gene	ID	Gene	ID	Gene	ID
*ABCA1*	rs2230806	*CRP*	rs1800947	*Ku70*	rs2267437
*ABCA1*	rs2249891	*CTLA4*	rs3087243	*Lig4*	rs1805386
*ABCB1*	rs1128503	*CXCR2*	rs1126579	*MDM2*	rs3730536
*ABCC1*	rs2230671	*CYC oxidase*	rs4646	*miR146A*	rs2910164
*ABCC1*	rs2981579	*Cyp2A6*	rs28399433	*miR187*	rs334348
*ABCC5*	rs7636910	*Cyp19A1*	rs51502844	*miR196A2*	rs11614913
*ATM*	rs1801516	*CZMB*	rs8192917	*miR206*	rs6920648
*ATM*	rs664143	*DNMT3B*	rs2424913	*miR34a*	rs4938723
*BB1/LPAR6*	rs2854344	*EGFR*	rs2293347	*MMP2*	rs243865
*BRCA1*	rs1799966	*EHBP1*	rs721048	*MTHFR*	rs1801133
*BRCA1*	rs799916	*ESR1*	rs2234693	*Nos3*	rs1799983
*BRCA2*	rs144848	*FAS/CD95*	rs2234978	*Nos3*	rs2070744
*Casp8*	rs1045485	*FGFR4*	rs2011077	*P21*	rs7767246
*Casp9*	rs1052576	*GSTP1*	rs1695	*PFA1*	rs10999426
*CCL2*	rs1024611	*HIFa1*	rs11549467	*PPAP2B*	rs1261411
*CCL2*	rs2530797	*HRas*	rs12628	*PRF1*	rs3758562
*CCL4*	rs1719153	*HTR3B*	rs3782025	*PRKDC*	rs1231204
*CCL5/Rantes*	rs2107538	*HTR3B*	rs1672717	*RaD52*	rs11571424
*CCL5/Rantes*	rs2280789	*IFNg*	rs2069705	*Serpin1*	rs1243168
*CCND1*	rs602652	*IFNg*	rs2069718	*STAT4*	rs7574865
*CCND3*	rs3218086	*IFNg RNA*	rs2430561	*TERT*	rs2736100
*CD44*	rs187115	*IGF1R*	rs951715	*TGFb*	rs1800469
*CD44*	rs7116432	*IL10*	rs1518111	*TNF*	rs1800610
*CDH13*	rs12445758	*IL12Rb2*	rs3790568	*TNF*	rs1800629
*CDKN2A*	rs3088440	*IL2*	rs6822844	*TNFA1P2*	rs8126
*CHARNA5*	rs16969968	*IL2/TRPC3*	rs11938795	*TNFSF1*	rs1054016
*Check2*	rs17879961	*IL2B*	rs3212227	*TP53*	rs1042522
*CHRNA3*	rs1051730	*IL2RA*	rs12722489	*Tyk2*	rs12720356
*CHRNA3*	rs10802789	*IL6*	rs1800797	*XRCC1*	rs25487
*COMT*	rs4680	*KDM4C*	rs2296067	*ZMF830*	rs3744355
*COMT1*	rs165722	*KDM4C*	rs818912		

All 92 SNP sequences were tested and passed two-hits in the dbSNP database and were Hap Map-validated with Illumina design ability score [19]. Genotyping of SNP was done at the SNP & SEQ Technology Platform, Uppsala University, Sweden (www.genotyping.se). High-throughput genotyping with the Illumina Golden gate assay was used according to the manufacturer’s protocol (www.illumina.com).

### Statistical analysis

Results are presented as numbers, means and standard deviations. Fishers exact test were used to evaluate possible differences in the frequency distribution of SNPs between the patient and control group. To examine the impact of cigarette smoking and SNPs, comparisons between SNP frequencies among the cigarette smoking (smoking) patients in each group to their corresponding smoking controls were analyzed. For the impact of unknown co-factors, SNP frequencies in patients who never had used any type of tobacco products (non-smoking) in each patient group were compared to non-smoking controls.

In the univariable logistic regression, the results were presented as Wald Chi-square test *p*-values, odds ratios (OR) and corresponding 95% confidence intervals (95% CI). The most frequent SNP among controls were used as reference level for OR in regression models. Stratified by disease and smoking status, no other interaction effects were analyzed.

Statistical analyses were done using SAS 9.4 software (SAS Institute, Cary, NC). All comparisons were two-sided and a Fishers exact test *p* value ≤0.05 was considered statistically significant.

## Results

### Controls and patients

A total of 512 participants were enrolled from one community based population in Jönköping region, Sweden during 2016 to 2019 (Table 1). There were 302 controls (155 males and 147 females). The median age of females was 55 years (range 20–89) and of males was 56 years (range 19–79). Among controls, 150 had a history of cigarette smoking (smoking) and 160 had never use any type of tobacco products (non-smoking).

A total 210 patients (154 males and 56 females) were included. The median age of females was 69 years (range 52–84) and of males was 71 years (range 39–91). The 98 CAD patients (83 males and 15 females) were 53 smoking and 45 non-smoking. The 74 UBCa patients (59 males and 15 females) were 15 smoking and 59 non-smoking. The 38 LCa patients (12 males and 26 females) were 31 smoking and 7 non-smoking.

### SNP distribution among CAD, UBCa and LCa patient groups and control group

Out of the 92 SNPs tested (Table 2), differences in distribution of 14 SNPs were detected between controls and the CAD, UBCa or LCa patients (Table 3). Differences between controls and CAD patients were detected in six SNPs located in *ATM*, *CTLA4*, *BRCA1*, *CCND3*, *HRas* and *IL2*. Differences between controls and UBCa patients were detected in six SNPs located in *ABCC5*, *CDH13*, *CRP*, *CTLA4*, *p2* and *TNFSF1* Differences between controls and LCa patients were detected in three SNPs located in *DNMT3B*, *MTHFR*, and *Serpin1*.

**Table 3:** SNP sequences of 302 controls (C), 98 cardiovascular artery disease (CAD), 74 urinary bladder cancer (UBCa) and 38 lung cancer (LCa) patients.

					Fisher exact test							Fisher exact test						
Gene	rs ID	Sequence	Control (n)	CAD (n)	p-value	Wald Chi^2^	Pr>Chi^2^	Odds Ratio	Estimate	95% Confidence Limits	UBCa (n)	p-value	Wald Chi^2^	Pr>Chi^2^	Odds Ratio	Estimate	95% Confidence Limits	LCa (n)
					C vs CAD							C vs UBCa						
*ABCC5*	rs7636910	AA	138	46	0.48	2.73	0.25	A/A vs A/G	1.18	0.73–1.92	37	0.02	7.48	0.02	A/A vs A/G	1.52	0.87–2.66	21
		AG	142	40				A/A vs G/G	0.61	0.28–1.33	25				A/A vs G/G	0.49	0.22–1.09	13
		GG	22	12				G/G vs A/G	1.93	0.88–4.25	12				G/G vs A/G	3.10	1.36–7.05	4
*ATM*	rs1801516	AA	6	6	0.05	5.36	0.07	A/A vs A/G	4.09	1.20–13.91	1	0.69	0.72	0.70	A/A vs A/G	0.79	0.09–6.94	0
		AG	90	22				A/A vs G/G	2.91	0.73–1.92	19				A/A vs G/G	0.63	0.07–5.34	12
		GG	204	70				A/G vs G/G	0.71	0.41–1.22	54				A/G vs G/G	0.79	0.45–1.42	26
*BRCA1*	rs1799966	AA	156	39	0.03	6.80	0.03	A/G vs A/A	1.46	0.90–2.36	39	0.22	0.94	0.63	A/G vs A/A	0.87	0.51–1.49	24
		AG	129	47				G/G vs A/A	2.82	1.24–6.39	28				G/G vs A/A	1.41	0.52–3.82	12
		GG	17	12				A/G vs G/G	0.52	0.23–1.16	6				A/G vs G/G	0.62	0.22–1.70	2
*CCND3*	rs3218086	AA	13	7	0.01	8.56	0.01	A/A vs A/G	1.08	0.39–2.92	1	0.28	2.33	0.31	A/A vs A/G	0.25	0.03–1.99	2
		AG	74	37				A/A vs G/G	2.14	0.82–5.63	23				A/A vs G/G	0.33	0.04–2.59	13
		GG	215	54				A/G vs G/G	1.99	1.21–3.27	50				A/G vs G/G	1.34	0.76–2.34	23
*CDH13*	rs12445758	AA	39	6	0.06	0.77	0.68	A/A vs A/G	1.28	0.65–2.53	16	0.02	7.46	0.02	A/A vs A/G	1.42	0.71–2.82	1
		AG	128	41				A/A vs G/G	1.35	0.69–2.66	37				A/A vs G/G	2.64	1.26–5.54	22
		GG	135	41				A/G vs G/G	1.06	0.64–1.73	21				A/G vs G/G	1.86	1.03–3.34	15
*CRP*	rs1800947	CC	261	86	0.62	0.88	0.64	C/G vs C/C	0.84	0.41–1.69	58	0.05	4.71	0.10	C/G vs C/C	1.58	0.80–3.08	30
		CG	40	11				G/G vs C/C	3.04	0.19–49.04	14				G/G vs C/C	9.00	0.80–100.93	7
		GG	1	1				C/G vs G/G	0.28	0.02–4.76	2				C/G vs G/G	0.18	0.02–2.08	1
*CTLA4*	rs3087243	AA	68	36	0.003	11.12	0.003	A/A vs A/G	1.50	0.86–2.61	29	0.004	10.79	0.005	A/A vs A/G	1.67	0.91–3.09	6
		AG	102	36				A/A vs G/G	2.69	1.50–4.82	16				A/A vs G/G	2.96	1.55–5.67	18
		GG	132	26				A/G vs G/G	1.79	1.02–3.16	19				A/G vs G/G	1.77	0.93–3.38	14
*DNMT3B*	rs2424913	AA	67	16	0.24	1.69	0.43	A/A vs A/G	0.69	0.37–1.31	15	0.92	0.16	0.92	A/A vs A/G	0.87	0.45–1.69	14
		AG	152	52				A/A vs G/G	0.65	0.32–1.28	39				A/A vs G/G	0.91	0.43–1.91	20
		GG	81	30				G/G vs A/G	1.08	0.64–1.83	20				G/G vs A/G	0.96	0.52–1.76	4
*Hras*	rs12628	AA	119	55	0.04	6.43	0.04	A/A vs A/G	1.78	1.09–2.92	33	0.74	0.59	0.74	A/A vs A/G	1.21	0.69–2.11	18
		AG	131	34				A/A vs G/G	1.95	0.88–4.32	30				A/A vs G/G	0.96	0.44–2.08	17
		GG	38	9				G/G vs A/G	0.91	0.40–2.07	11				G/G vs A/G	1.26	0.58–2.76	3
*IL2*	rs6822844	AA	15	2	0.04	6.02	0.05	A/A vs A/C	0.59	0.13–2.75	5	0.61	0.98	0.61	A/A vs A/C	1.61	0.53–4.88	0
		AC	126	24				A/A vs C/C	0.34	0.08–1.50	22				A/A vs C/C	1.28	0.44–3.71	10
		CC	81	72				C/C vs A/C	1.76	1.04–2.96	47				C/C vs A/C	1.25	0.72–2.19	28
*MTHFR*	rs1801133	AA	20	11	0.26	2.60	0.27	A/A vs A/G	1.97	0.86–4.52	10	0.14	3.77	0.15	A/A vs A/G	2.10	0.89–4.97	7
		AG	122	34				A/A vs G/G	1.66	0.74–3.69	29				A/A vs G/G	2.29	0.98–5.31	15
		GG	160	53				A/G vs G/G	0.84	0.52–1.38	35				A/G vs G/G	1.09	0.63–1.88	16
*P21*	rs7767246	CC	209	70	0.51	1.30	0.52	C/G vs C/C	0.83	0.05–1.41	40	0.05	6.05	0.04	C/G vs C/C	1.88	1.11–3.21	26
		CG	86	24				G/G vs C/C	1.71	0.48–6.01	31				G/G vs C/C	2.24	0.56–9.03	9
		GG	7	4				C/G vs G/G	0.49	0.13–1.81	3				C/G vs G/G	0.84	0.21–3.46	3
*Serpin 1*	rs1243168	AA	21	7	0.82	0.40	0.82	A/A vs A/G	1.13	0.44–2.88	2	0.37	1.86	0.39	A/A vs A/G	0.39	0.09–1.77	0
		AG	115	34				A/A vs G/G	0.97	0.39–2.39	28				A/A vs G/G	0.36	0.08–1.58	8
		GG	165	57				A/G vs G/G	0.86	0.53–1.39	44				A/G vs G/G	0.91	0.54–1.55	30
*TNFSF1*	rs1054016	AA	65	18	0.48	1.48	0.48	A/A vs A/C	0.69	0.37–1.26	9	0.02	7.21	0.03	A/A vs A/C	0.50	0.23–1.11	7
		AC	131	53				A/A vs C/C	0.76	0.38–1.53	36				A/A vs C/C	0.33	0.15–0.75	17
		CC	69	25				C/C vs A/C	0.90	0.51–1.56	29				C/C vs A/C	1.53	0.87–2.70	14
					Fisher exact test													
Gene	rs ID	Sequence	Control (n)	CAD (n)	LCa (n)	p-value	Wald Chi^2^	Pr>Chi^2^	Odds Ratio	Estimate	95% Confidence Limits							
						C vs LCa												
*ABCC5*	rs7636910	AA	138	46	21	0.31	2.30	0.32	A/A vs A/G	1.66	0.80–3.45							
		AG	142	40	13				A/A vs G/G	0.84	0.26–2.67							
		GG	22	12	4				G/G vs A/G	1.99	0.59–6.64							
*ATM*	rs1801516	AA	6	6	0	0.85	1.459*	0.48	A/A vs A/G	N/A	N/A							
		AG	90	22	12				A/A vs G/G	N/A	N/A							
		GG	204	70	26				A/G vs G/G	1.05	0.51–2.17							
*BRCA1*	rs1799966	AA	156	39	24	0.39	1.83	0.39	A/G vs A/A	0.61	0.29–1.26							
		AG	129	47	12				G/G vs A/A	0.77	0.16–3.52							
		GG	17	12	2				A/G vs G/G	0.79	0.16–3.84							
*CCND3*	rs3218086	AA	13	7	2	0.29	1.84	0.39	A/A vs A/G	0.88	0.17–4.34							
		AG	74	37	13				A/A vs G/G	1.44	0.30–6.77							
		GG	215	54	23				A/G vs G/G	1.64	0.79–3.40							
*CDH13*	rs12445758	AA	39	6	1	0.08	4.29	0.12	A/A vs A/G	0.15	0.01–1.14							
		AG	128	41	22				A/A vs G/G	0.23	0.03–1.80							
		GG	135	41	15				A/G vs G/G	1.55	0.76–3.11							
*CRP*	rs1800947	CC	261	86	30	0.14	3.01	0.22	C/G vs C/C	1.52	0.62–3.69							
		CG	40	11	7				G/G vs C/C	8.70	0.53–142.70							
		GG	1	1	1				C/G vs G/G	0.18	0.01–3.13							
*CTLA4*	rs3087243	AA	68	36	6	0.24	2.78	0.25	A/A vs A/G	0.50	0.18–1.32							
		AG	102	36	18				A/A vs G/G	0.83	0.30–2.26							
		GG	132	26	14				A/G vs G/G	1.66	0.79–3.50							
*DNMT3B*	rs2424913	AA	67	16	14	0.04	6.06	0.05	A/A vs A/G	1.59	0.75–3.33							
		AG	152	52	20				A/A vs G/G	4.23	1.33–13.45							
		GG	81	30	4				G/G vs A/G	0.37	0.12–1.13							
*Hras*	rs12628	AA	119	55	18	0.59	1.02	0.59	A/A vs A/G	1.16	0.57–2.36							
		AG	131	34	17				A/A vs G/G	1.91	0.53–6.86							
		GG	38	9	3				G/G vs A/G	0.61	0.16–2.18							
*IL2*	rs6822844	AA	15	2	0	0.15	1.62*	0.44	A/A vs A/C	N/A	N/A							
		AC	126	24	10				A/A vs C/C	N/A	N/A							
		CC	81	72	28				C/C vs A/C	1.64	0.77–3.51							
*MTHFR*	rs1801133	AA	20	11	7	0.03	6.12	0.05	A/A vs A/G	2.85	1.03–7.85							
		AG	122	34	15				A/A vs G/G	3.50	1.28–9.54							
		GG	160	53	16				A/G vs G/G	1.23	0.58–2.58							
*P21*	rs7767246	CC	209	70	26	0.15	3.42	0.18	C/G vs C/C	0.84	0.37–1.86							
		CG	86	24	9				G/G vs C/C	3.45	0.83–14.15							
		GG	7	4	3				C/G vs G/G	0.24	0.05–1.11							
*Serpin 1*	rs1243168	AA	21	7	0	0.01	5.33*	0.07	A/A vs A/G	N/A	N/A							
		AG	115	34	8				A/A vs G/G	N/A	N/A							
		GG	165	57	30				A/G vs G/G	0.38	0.17–0.87							
*TNFSF1*	rs1054016	AA	65	18	7	0.35	2.05	0.36	A/A vs A/C	0.83	0.32–2.10							
		AC	131	53	17				A/A vs C/C	0.53	0.20–1.39							
		CC	69	25	14				C/C vs A/C	1.56	0.72–3.36							

*N/A not estimable due to zero or few individuals.

Of the 92 SNPs (Table 2), only *CTLA4* rs3087243 showed a statistically significant difference in two groups of the patients, CAD and UBCa, compared to the controls (Table 3).

### SNP distribution among smoking CAD, UBCa or LCa patients and smoking controls

Out of these 92 SNPs, differences in the distribution of 15 SNPs were detected between smoking controls and smoking patients (Table 4). Differences in seven SNPs located in *CCND3*, *CTLA4*, *KDM4C*, *PFA1*, *PPAP2B*, *PRF1* and *Rad52*, were found to be specific for smoking CAD patients. Differences in three SNPs located in *ABCA1*, *CCND1* and *MiR206* were specific for smoking UBCa patients. Differences in four SNPs located in *CDH13*, *HTR3B1*, *CRP* and *ZMF830* were specific for smoking LCa patients.

**Table 4:** Cigarette smoking and SNPs sequences of 142 controls (C), 55 cardiovascular artery disease (CAD), 15 urinary bladder cancer (UBCa) and 31 lung cancer (LCa) patients.

					Fisher exact test							Fisher exact test						
Gene	ID	Sequence	Control (n)	CAD (n)	p-value	Wald Chi^2^	Pr>Chi^2^	Odds Ratio	Estimate	95% Confidence Limits	UBCa (n)	p-value	Wald Chi^2^	Pr>Chi^2^	Odds Ratio	Estimate	95% Confidence Limits	
					C vs CAD							C vs UBCa						LCa (n)
*ABCA1*	rs2230806	AA	13	4	0.55	1.18	0.55	A/A vs A/G	0.95	0.27–3.30	1	0.03	0.57	0.75	A/A vs A/G	1.70	0.29–9.99	0
		AG	56	18				A/A vs G/G	0.68	0.20–2.24	1				A/A vs G/G	1.90	0.35–10.16	13
		GG	73	33				A/G vs G/G	0.71	0.36–1.39	13				A/G vs G/G	1.11	0.34–3.61	18
*CCND1*	rs602652	AA	21	12	0.07	5.09	0.07	A/G vs A/A	2.95	1.15–7.56	1	0.03	5.06	0.07	A/G vs A/A	11.81	1.35–103.01	10
		*AG*	16	27				G/G vs A/A	1.86	0.68–5.07	9				G/G vs A/A	6.99	0.74–66.19	13
		*GG*	15	16				A/G vs G/G	1.58	0.62–4.03	5				A/G vs G/G	1.68	0.46–6.19	8
*CCND3*	rs3218086	AA	8	4	0.01	9.09	0.01	A/A vs A/G	0.65	0.17–2.43	1	0.53	1.23	0.54	A/A vs A/G	0.75	0.07–7.36	1
		AG	30	23				A/A vs G/G	1.85	0.52–6.61	9				A/A vs G/G	1.44	0.16–12.87	11
		GG	104	28				A/G vs G/G	2.84	1.43–5.64	5				A/G vs G/G	1.92	0.60–6.18	19
*CDH13*	rs12445758	AA	15	6	0.79	0.45	0.79	A/A vs A/G	0.91	0.31–2.65	4	0.07	4.81	0.08	A/A vs A/G	1.90	0.49–7.40	1
		AG	50	22				A/A vs G/G	1.14	0.40–3.23	7				A/A vs G/G	5.13	1.15–22.82	18
		GG	77	27				A/G vs G/G	1.25	0.64–2.44	4				A/G vs G/G	2.69	0.75–9.68	12
*CRP*	rs1800947	CC	124	49	0.73	0.11*	0.73	C/G vs C/C	0.84	0.31–2.25	14	0.50	0.44*	0.50	C/G vs C/C	0.49	0.06–3.97	24
		CG	18	6				G/G vs C/C	N/A	N/A	1				G/G vs C/C	N/A	N/A	6
		GG	0	0				C/G vs G/G	N/A	N/A	0				C/G vs G/G	N/A	N/A	1
*CTLA4*	rs3087243	AA	35	24	0.03	6.02	0.04	A/A vs A/G	0.97	0.42–2.22	6	0.35	2.00	0.36	A/A vs A/G	1.57	0.44–5.59	5
		AG	46	15				A/A vs G/G	2.58	1.01–6.57	5				A/A vs G/G	2.61	0.69–9.90	13
		GG	61	16				A/G vs G/G	2.66	1.16–6.11	4				A/G vs G/G	1.65	0.42–6.51	13
*HTR3B*	rs1672717	AA	40	22	0.45	2.54	0.27	A/A vs A/G	1.69	0.85–3.35	2	0.77	1.44	0.48	A/A vs A/G	0.40	0.08–1.91	14
		AG	80	126				A/A vs G/G	1.72	0.63–4.68	10				A/A vs G/G	0.36	0.05–2.36	10
		GG	22	7				G/G vs A/G	0.97	0.37–2.55	3				G/G vs A/G	1.09	0.27–4.30	7
*KDM4C*	rs2296067	AA	5	3	0.01	8.58	0.01	A/A vs A/G	0.94	0.20–4.22	1	0.76	0.53	0.76	A/A vs A/G	2.35	0.21–25.32	0
		AG	47	30				A/A vs G/G	2.45	0.54–11.05	4				A/A vs G/G	1.80	0.19–16.98	15
		GG	90	22				A/G vs G/G	2.61	1.35–5.01	10				A/G vs G/G	0.76	0.22–2.57	16
*miR206*	rs6920648	AA	41	13	0.74	0.58	0.74	A/A vs A/G	0.74	0.35–1.58	9	0.03	5.94	0.05	A/A vs A/G	5.34	1.36–20.84	9
		AG	73	31				A/A vs G/G	0.80	0.31–2.05	3				A/A vs G/G	2.04	0.50–8.24	17
		GG	28	11				G/G vs A/G	0.92	0.41–2.08	3				G/G vs A/G	2.60	0.49–13.69	5
*PFA1*	rs10999426	AA	10	6	0.02	3.73	0.15	A/A vs A/G	0.18	0.02–1.48	2	0.47	1.47	0.47	A/A vs A/G	2.60	0.46–14.67	4
		AG	78	18				A/A vs G/G	0.30	0.03–2.43	6				A/A vs G/G	1.54	0.27–8.53	14
		GG	54	31				G/G vs A/G	0.62	0.30–1.26	7				G/G vs A/G	1.68	0.53–5.29	13
*PPAP2B*	rs1261411	AA	18	2	0.04	5.88	0.05	A/A vs A/G	0.20	0.04–0.92	0	0.26	0.44*	0.79	A/A vs A/G	N/A	N/A	5
		AG	62	34				A/A vs G/G	0.35	0.07–1.65	9				A/A vs G/G	N/A	N/A	18
		GG	60	29				G/G vs A/G	0.58	0.29–1.12	6				G/G vs A/G	0.68	0.23–2.05	8
*PRF1*	rs3758562	AA	53	30	0.03	3.53	0.17	A/A vs A/G	0.64	0.31–1.29	7	0.44	1.56	0.45	A/A vs A/G	1.73	0.55–5.46	13
		AG	79	19				A/A vs G/G	3.38	0.41–27.48	6				A/A vs G/G	0.66	0.12–3.65	14
		GG	10	6				G/G vs A/G	0.19	0.02–1.50	2				G/G vs A/G	2.63	0.46–14.85	4
*RAD52*	rs11571424	AA	0	2	0.01	0.33	0.84	A/A vs A/G	1.46	0.13–15.61	0	0.43	0.60	0.43	A/A vs A/G	N/A	N/A	1
		AG	26	17				A/A vs G/G	1.17	0.11–11.70	4				A/A vs G/G	N/A	N/A	4
		GG	26	36				G/G vs A/G	1.24	0.56–2.74	11				G/G vs A/G	0.61	0.18–2.09	26
*ZMF830*	rs3744355	CC	125	46	0.71	1.46*	0.47	C/G vs C/C	0.53	0.19–1.47	12	0.51	1.09*	0.57	C/G vs C/C	2.08	0.52–8.23	22
		CG	15	8				G/G vs C/C	N/A	N/A	3				G/G vs C/C	N/A	N/A	8
		GG	2	1				C/G vs G/G	N/A	N/A	0				C/G vs G/G	N/A	N/A	1
					Fisher exact test													
Gene	ID	Sequence	Control (n)	CAD (n)	p-value	Wald Chi^2^	Pr>Chi^2^	Odds Ratio	Estimate	95% Confidence Limits								
					C vs LCa													
*ABCA1*	rs2230806	AA	13	4	0.61	0.93	0.62	A/A vs A/G	0.35	0.04–3.03								
		AG	56	18				A/A vs G/G	0.42	0.05–3.49								
		GG	73	33				A/G vs G/G	1.19	0.52–2.68								
*CCND1*	rs602652	AA	21	12	0.58	1.09	0.57	A/G vs A/A	1.70	0.59–4.87								
		*AG*	16	27				G/G vs A/A	1.12	0.35–3.50								
		*GG*	15	16				A/G vs G/G	1.52	0.49–4.70								
*CCND3*	rs3218086	AA	8	4	0.22	2.92	0.23	A/A vs A/G	0.34	0.03–3.04								
		AG	30	23				A/A vs G/G	0.68	0.08–5.79								
		GG	104	28				A/G vs G/G	2.00	0.86–4.67								
*CDH13*	rs12445758	AA	15	6	0.04	5.64	0.05	A/A vs A/G	0.18	0.02–1.50								
		AG	50	22				A/A vs G/G	0.42	0.05–3.54								
		GG	77	27				A/G vs G/G	2.31	1.02–5.20								
*CRP*	rs1800947	CC	124	49	0.05	1.08*	0.58	C/G vs C/C	1.72	0.62–4.78								
		CG	18	6				G/G vs C/C	N/A	N/A								
		GG	0	0				C/G vs G/G	N/A	N/A								
*CTLA4*	rs3087243	AA	35	24	0.48	1.45	0.48	A/A vs A/G	0.50	0.16–1.55								
		AG	46	15				A/A vs G/G	0.67	0.22–2.03								
		GG	61	16				A/G vs G/G	1.32	0.56–3.13								
*HTR3B*	rs1672717	AA	40	22	0.05	5.67	0.05	A/A vs A/G	2.80	1.14–6.85								
		AG	80	126				A/A vs G/G	1.10	0.38–3.13								
		GG	22	7				G/G vs A/G	2.54	0.86–7.45								
*KDM4C*	rs2296067	AA	5	3	0.19	2.12*	0.34	A/A vs A/G	N/A	N/A								
		AG	47	30				A/A vs G/G	N/A	N/A								
		GG	90	22				A/G vs G/G	1.79	0.81–3.94								
*miR206*	rs6920648	AA	41	13	0.89	0.22	0.89	A/A vs A/G	0.94	0.38–2.30								
		AG	73	31				A/A vs G/G	1.22	0.37–4.05								
		GG	28	11				G/G vs A/G	0.76	0.25–2.27								
*PFA1*	rs10999426	AA	10	6	0.44	1.59	0.45	A/A vs A/G	2.22	0.61–8.10								
		AG	78	18				A/A vs G/G	1.66	0.44–6.14								
		GG	54	31				G/G vs A/G	1.34	0.58–3.07								
*PPAP2B*	rs1261411	AA	18	2	0.21	2.99	0.22	A/A vs A/G	0.95	0.31–2.93								
		AG	62	34				A/A vs G/G	2.08	0.60–7.15								
		GG	60	29				G/G vs A/G	0.45	0.18–1.13								
*PRF1*	rs3758562	AA	53	30	0.42	1.69	0.42	A/A vs A/G	1.38	0.60–3.17								
		AG	79	19				A/A vs G/G	0.61	0.16–2.27								
		GG	10	6				G/G vs A/G	2.25	0.62–8.21								
*RAD52*	rs11571424	AA	0	2	0.08	0.42*	0.80	A/A vs A/G	N/A	N/A								
		AG	26	17				A/A vs G/G	N/A	N/A								
		GG	26	36				G/G vs A/G	1.45	0.46–4.53								
*ZMF830*	rs3744355	CC	125	46	0.05	5.43	0.06	C/G vs C/C	3.03	1.14–7.99								
		CG	15	8				G/G vs C/C	2.84	0.24–32.68								
		GG	2	1				C/G vs G/G	1.06	0.08–13.65								

*N/A not estimable due to zero or few individuals.

None of these 92 SNPs showed a statistically significant difference in distribution in more than one disease among smoking patients compared to their corresponding smoking controls (Table 4).

### SNPs distribution among non-smoking CAD, UBCa, LCa patients and non-smoking controls

Out of these 92 SNPs tested, differences in distribution of 14 SNPs were detected between the non-smoking controls and non-smoking patients (Table 5). They were seven SNPs located in ATM, BRCA1, *CTLA4*, CYP19A1, FGFR4, *MiR34A* and *PRKDC* in non-smoking CAD patients. Three SNPs located in *MTHFR*, *CTLA4*, *and XRCC1*, in non-smoking UBCa patients and four SNPs located in *HTRB1*, *Lig4*, *Serpin1*, *TGFb*, and *XRCC1* in non-smoking LCa patients (Table 5).

**Table 5:** Non-smoking and SNP sequences of 160 controls (C), 43 cardiovascular artery disease (CAD), 59 urinary bladder cancer (UBCa) and 7 lung cancer (LCa) patients.

					Fisher exact test							Fisher exact test					
Gene	ID	Sequence	Control (n)	CAD (n)	p-value	Wald Chi^2^	Pr>Chi^2^	Odds Ratio	Estimate	95% Confidence Limits	UBCa (n)	p-value	Wald Chi^2^	Pr>Chi^2^	Odds Ratio	Estimate	95% Confidence Limits
					C vs CAD							C vs UBCa					
ATM	rs1801516	AA	1	3	0.02	4.90	0.08	A/A vs A/G	14.39	1.35–153.03	1	0.71	0.65	0.72	A/A vs A/G	2.99	0.17–50.74
		AG	48	10				A/A vs G/G	10.99	1.10–109.58	16				A/A vs G/G	2.61	0.16–42.79
		GG	110	30				A/G vs G/G	0.76	0.34–1.68	42				A/G vs G/G	0.87	0.44–1.70
BRCA1	rs1799966	AA	87	15	0.02	6.92	0.03	A/G vs A/A	1.96	0.94–4.07	29	0.57	1.07	0.58	A/G vs A/A	1.10	0.59–2.07
		AG	65	22				G/G vs A/A	4.35	1.32–14.32	24				G/G vs A/A	1.87	0.56–6.18
		GG	8	6				A/G vs G/G	0.45	0.14–1.44	5				A/G vs G/G	0.59	0.17–1.98
CTLA4	rs3087243	AA	33	12	0.04	6.02	0.04	A/A vs A/G	0.97	0.42–2.22	23	0.01	9.24	0.01	A/A vs A/G	1.85	0.89–3.86
		AG	56	21				A/A vs G/G	2.58	1.01–6.57	21				A/A vs G/G	3.29	1.52–7.13
		GG	71	10				A/G vs G/G	2.66	1.16–6.10	15				A/G vs G/G	1.77	0.83–3.75
CYP19A1	rs51502844	AA	5	7	0.02	7.32	0.02	A/A vs A/C	5.46	1.42–10.88	4	0.85	0.32	0.85	A/A vs A/C	1.30	0.31–5.32
		AC	39	10				A/A vs C/C	2.26	0.65–7.87	24				A/A vs C/C	1.08	0.26–4.37
		CC	42	26				A/C vs C/C	0.41	0.17–0.96	31				A/C vs C/C	0.83	0.41–1.66
EHBP1	rs721048	AA	4	2	0.19	3.22	0.19	A/A vs A/G	1.35	0.22–7.95	2	0.81	0.41	0.81	A/A vs A/G	1.22	0.20–7.19
		AG	54	20				A/A vs G/G	2.42	0.41–14.13	22				A/A vs G/G	1.45	0.25–8.30
		GG	102	21				A/G vs G/G	1.79	0.89–3.60	35				A/G vs G/G	1.18	0.63–2.22
FGFR4	rs2011077	AA	8	0	0.04	3.61*	0.16	A/A vs A/G	N/A	N/A	2	0.53	1.26	0.53	A/A vs A/G	0.54	0.10–2.76
		AG	50	21				A/A vs G/G	N/A	N/A	23				A/A vs G/G	0.75	0.15–3.70
		GG	102	22				A/G vs G/G	1.94	0.98–3.87	34				A/G vs G/G	1.38	0.73–2.58
HTR3B1	rs3782025	AA	44	11	0.92	0.06	0.96	A/A vs A/G	0.91	0.40–2.07	15	0.71	1.87	0.39	A/A vs A/G	0.77	0.37–1.57
		AG	77	21				A/A vs G/G	0.88	0.34–2.27	34				A/A vs G/G	1.32	0.53–3.29
		GG	39	11				G/G vs A/G	1.03	0.45–2.36	0				G/G vs A/G	0.58	0.26–1.29
Lig4	rs1805386	AA	120	29	0.46	1.49	0.47	A/G vs A/A	1.58	0.74–3.37	45	0.37	0.06*	0.96	A/G vs A/A	1.09	0.54–2.23
		AG	34	13				G/G vs A/A	0.82	0.09–7.35	14				G/G vs A/A	N/A	N/A
		GG	5	1				A/G vs G/G	1.91	0.20–17.95	0				A/G vs G/G	N/A	N/A
miR34A	rs4938723	AA	63	17	0.03	6.60	0.03	A/A vs A/G	1.42	0.65–3.06	24	0.89	0.23	0.89	A/A vs A/G	1.00	0.53–1.88
		AG	79	15				A/A vs G/G	0.41	0.16–1.05	30				A/A vs G/G	1.29	0.43–3.90
		GG	17	11				G/G vs A/G	3.40	1.33–8.70	5				G/G vs A/G	0.77	0.26–2.28
MTHFR	rs1801133	AA	8	4	0.45	1.57	0.45	A/A vs A/G	2.30	0.60–8.82	10	0.01	9.14	0.01	A/A vs A/G	3.00	1.06–8.48
		AG	60	13				A/A vs G/G	1.76	0.49–6.34	25				A/A vs G/G	4.79	1.70–13.45
		GG	92	26				A/G vs G/G	0.76	0.36–1.60	24				A/G vs G/G	1.59	0.83–3.05
PRKDC	rs1231204	CC	0	2	0.01	1.60*	0.44	C/C vs C/G	N/A	N/A	0	0.15	1.97	0.15	C/C vs C/G	N/A	N/A
		CG	29	4				C/C vs G/G	N/A	N/A	6				C/C vs G/G	N/A	N/A
		GG	131	37				C/G vs G/G	0.48	0.16–1.47	53				C/G vs G/G	0.51	0.20–1.30
Serpin1	rs1243168	AA	12	4	0.86	0.31	0.85	A/A vs A/G	1.16	0.33–4.06	2	0.41	1.71	0.42	A/A vs A/G	0.50	0.10–2.41
		AG	63	18				A/A vs G/G	1.34	0.39–4.60	21				A/A vs G/G	0.39	0.08–1.84
		GG	85	21				A/G vs G/G	1.15	0.56–2.35	36				A/G vs G/G	0.78	0.42–1.47
TGFb	rs1800469	AA	18	3	0.32	2.98	0.22	A/A vs A/G	0.44	0.12–1.66	6	0.39	1.84	1.84	A/A vs A/G	0.71	0.25–1.98
		AG	62	23				A/A vs G/G	0.77	0.20–2.92	29				A/A vs G/G	1.09	0.39–3.07
		GG	79	17				A/G vs G/G	1.72	0.84–3.50	24				A/G vs G/G	1.54	0.81–2.90
XRCC1	rs25487	AA	17	4	0.78	0.48	0.78	A/A vs A/G	0.98	0.29–3.33	11	0.05	5.70	0.05	A/A vs A/G	1.44	0.60–3.45
		AG	67	16				A/A vs G/G	0.77	0.23–2.54	30				A/A vs G/G	2.73	1.09–6.82
		GG	76	23				A/G vs G/G	0.78	0.38–1.61	18				A/G vs G/G	1.89	0.96–3.69
					Fisher exact test												
Gene	ID	Sequence	Control (n)	CAD (n)	LCa (n)	p-value	Wald Chi^2^	Pr>Chi^2^	Odds Ratio	Estimate	95% Confidence Limits						
						C vs LCa											
ATM	rs1801516	AA	1	3	0	0.64	0.77*	0.68	A/A vs A/G	N/A	N/A						
		AG	48	10	1				A/A vs G/G	N/A	N/A						
		GG	110	30	6				A/G vs G/G	0.38	0.04–3.25						
BRCA1	rs1799966	AA	87	15	0	0.20	1.88*	0.39	A/G vs A/A	0.22	0.02–1.89						
		AG	65	22	1				G/G vs A/A	N/A	N/A						
		GG	8	6	6				A/G vs G/G	N/A	N/A						
CTLA4	rs3087243	AA	33	12	1	0.14	3.23	0.19	A/A vs A/G	0.33	0.03–3.03						
		AG	56	21	5				A/A vs G/G	2.15	0.13–35.46						
		GG	71	10	1				A/G vs G/G	6.33	0.72–55.82						
CYP19A1	rs51502844	AA	5	7	1	0.24	2.34	0.31	A/A vs A/C	7.80	0.41–145.19						
		AC	39	10	1				A/A vs C/C	1.68	0.16–17.41						
		CC	42	26	5				A/C vs C/C	0.22	0.02–1.92						
EHBP1	rs721048	AA	4	2	0	0.02	4.93*	0.08	A/A vs A/G	N/A	N/A						
		AG	54	20	6				A/A vs G/G	N/A	N/A						
		GG	102	21	1				A/G vs G/G	11.33	1.33–96.57						
FGFR4	rs2011077	AA	8	0	0	0.33	1.63*	0.44	A/A vs A/G	N/A	N/A						
		AG	50	21	4				A/A vs G/G	N/A	N/A						
		GG	102	22	3				A/G vs G/G	2.72	0.58–12.62						
HTR3B1	rs3782025	AA	44	11	1	0.021	5.66	0.05	A/A vs A/G	1.75	0.10–28.67						
		AG	77	21	1				A/A vs G/G	0.17	0.02–1.58						
		GG	39	11	5				G/G vs A/G	9.87	1.11–87.44						
Lig4	rs1805386	AA	120	29	2	0.01	6.42*	0.04	A/G vs A/A	8.82	1.63–47.51						
		AG	34	13	5				G/G vs A/A	N/A	N/A						
		GG	5	1	0				A/G vs G/G	N/A	N/A						
miR34A	rs4938723	AA	63	17	4	0.12	3.21	0.21	A/A vs A/G	N/A	N/A						
		AG	79	15	1				A/A vs G/G	N/A	N/A						
		GG	17	11	2				G/G vs A/G	N/A	N/A						
MTHFR	rs1801133	AA	8	4	0	0.20	2.48*	0.29	A/A vs A/G	N/A	N/A						
		AG	60	13	5				A/A vs G/G	N/A	N/A						
		GG	92	26	2				A/G vs G/G	3.83	0.72–20.31						
PRKDC	rs1231204	CC	0	2	0	0.21	0.00*	0.96	C/C vs C/G	N/A	N/A						
		CG	29	4	0				C/C vs G/G	N/A	N/A						
		GG	131	37	7				C/G vs G/G	N/A	N/A						
Serpin1	rs1243168	AA	12	4	0	0.05	0.01*	0.99	A/A vs A/G	N/A	N/A						
		AG	63	18	0				A/A vs G/G	N/A	N/A						
		GG	85	21	7				A/G vs G/G	N/A	N/A						
TGFb	rs1800469	AA	18	3	1	0.03	0.25*	0.88	A/A vs A/G	0.57	0.07–5.08						
		AG	62	23	6				A/A vs G/G	N/A	N/A						
		GG	79	17	0				A/G vs G/G	N/A	N/A						
XRCC1	rs25487	AA	17	4	3	0.04	5.23	0.07	A/A vs A/G	5.91	0.91–38.23						
		AG	67	16	2				A/A vs G/G	6.71	1.04–43.29						
		GG	76	23	2				A/G vs G/G	1.13	0.16–8.28						

*N/A not estimable due to zero or few individuals.

Two of these 92 SNPs, *CTLA*4 *rs3087243* and XRCC1 *rs25487* showed different distribution in more than one disease among non-smoking patient groups compared to their non-smoking controls (Table 5). The SNP in *CTLA*4 rs3087243 showed a difference in distribution among non-smoking controls and non-smoking CAD or UBCa patients. The SNP in *XRCC*1 rs25487 showed a difference in distribution among non-smoking controls and non-smoking UBCa or LCa patients.

## Discussion

The genetic and the environment influence human risk of various diseases and clinical outcome. We found that specific genetic variations influenced the risk of diseases that are suggested to associate to cigarette smoking such as CVD, UBCa and LCa. These diseases are heterogeneous groups regarding site location and their pathological parameters.

Endothelial malignancies are very rare in the western population and consists mainly of sarcomas. The endothelial cells seems not to be transformed into malignancies from smoking [20]. However, the blockage of the CAD patient artery caused by smoking is a complex mechanism involving inflammation and benign smooth muscle proliferation [21, 22].

Out of these 92 SNPs, only distribution of *CTLA4* rs3087243 differed between controls and two patient groups, CAD and UBCa. *CTLA4* located on chromosome 2q33.2, encodes for CTLA4 protein that functions as an immune checkpoint and downregulates immune responses [23, 24]. CTLA4 is constitutively expressed in regulatory T cells. Upon activation, conventional T cells in cancer patients could up-regulated CTLA4 [25]. Alteration in CLTA4 expression cells were detected in the inflammatory heart disease [26]. *CTLA4* polymorphism and risk of cancer were reported [27, 28]. The possible benefits of immunotherapy with focus on CTLA4 protein as target therapy of CAD and UBCa need further investigation [25, 29, 30].

The impact of cigarette smoking on CAD risk was more pronounced among individuals with specific SNPs in genes involving the immune response *(CTLA4*, *PFA and PRF)*, cell cycle control *(CCND3* and *p21)*, DNA repair (*BRCA1*, *ATM*) and oncogene (*HRas*). The impact of cigarette smoking on UBCa risk seem to be accumulated among individuals with SNPs in genes involved in cell cycle control (*ABCC1*, *CDH13* and *p21*,) and immune response (*CRP*, *CTLA4* and *TNFSF1*). The impact of cigarette smoking on LCa risk might be increased among individuals with SNPs in genes involving cell cycle control (*CDH13* and *HTR3B1*) and immune response (*CRP*). The variation in SNPs among cigarette smoking CAD, UBCa and LCa patients indicates an impact of general toxic agents from cigarette smoke and specific DNA sequences, not random event on the risk for these diseases.

There are subgroups of the CAD, UBCa and LCa patients that never have used any type of tobacco products, non-smoking patient group. Despite similar type of disease, SNPs that associated to the non-smoking UBCa patients differed from those of the smoking UBCa patients. Unknown risk factors, apart from cigarette smoking, in combination with the specific SNPs could also increase the risk of UBCa in non-smoking individuals.

SNP sequence variation in *CTLA4* rs3087243 was associated to risk of CAD in both smoking and non-smoking patients. Thus, the influence of immune response on smooth muscle cell proliferation of CAD patients [26] was independent from cigarette smoking. SNPs in the *CTLA4* rs3087243 also associate to risk of non- smoking UBCa patients, not in smoking UBCa patients. This suggests an influence of *CTLA4* rs3087243 on risk of CAD and UBCa might be independent from the cigarette smoke.

*XRCC1* gene located on chromosome 19q13.2, encoded for XRCC1 protein is an essential for DNA damage repair [31–33]. Genetic variations that influence DNA damage repair efficiency in combination with harm micro-environment might increase risk of UBCa and LCa [34–36].

The differences in *XRCC1* rs25487 of non-smoking UBCa and non-smoking LCa patients compared to non-smoking controls, confirms that DNA repair play an important role on UBCa and LCa [34, 35]. If patients with AA or AG sequences in *XRCC1* rs25487 are prone to DNA damage from other agents/microenvironments more than cigarette smoke needs further investigation [37–39].

Our pilot study has several limitations. Firstly, this is a single center, non- randomized retrospective study with a relatively low number of included patients [40]. In addition, the lower age among controls compared to patients was based on the blood bank regulation, needs to be considered. Secondly, cigarette smoking history of the patients and controls was self-reported and this could be influenced by recall bias [41]. Thirdly, passive smoking conditions that might have impact on risk of these diseases [8, 42], were not recorded.

In summary, our results indicate an important role of specific genetic variations on risk of CAD, UBCa or LCa. Impact of cigarette smoking was also found in a proportion of these patients in association with individual specific SNPs. The SNPs are lifelong stable genetic variation that could predict the risk to develop specific diseases.

Unable to quit smoking during or after treatment could increase rate of diseases recurrence, progression, development of a second primary tumour and disease-specific mortality. The identification of SNPs that associated to risk or disease progression could increase the cessation rate in smoking patients.

Healthy individual with SNPs associated to a smoking related risk to develop CAD, UBCa or LCa will also get higher motivation for smoking cessation. Unknown environmental factors associated to risk of these diseases in non-smoking group [43] and the possibility to use SNPs as prognostic biomarkers for treatment selection and prediction of clinical outcome needs future investigation.
